# Does marriage work as a savings commitment device? Experimental evidence from Vietnam

**DOI:** 10.1371/journal.pone.0217646

**Published:** 2019-06-19

**Authors:** Hisaki Kono, Tomomi Tanaka

**Affiliations:** 1 Graduate School of Economics, Kyoto University, Kyoto, Japan; 2 World Bank, Washington, D.C., United States of America; Universidad Loyola Andalucia, SPAIN

## Abstract

Present bias, or the overvaluation of an immediate payoff, causes under-saving and financial difficulty. We investigate whether married couples utilize their spouses as a savings commitment device to alleviate the present bias problem using experimental and survey data in Vietnam. We find that individuals are less present biased when making joint decisions with their spouses than they are when making decisions alone. However, present-biased individuals turn over a smaller ratio of their earnings to their spouses and are more likely to manage household resources than time-consistent individuals are. Present-biased individuals also receive larger amounts of money from their spouses’ incomes, indicating that marriage not only fails to function as a savings commitment device but also exacerbates the problem. Married couples whose joint decisions are not present biased try to alleviate this problem by allocating smaller allowances to present-biased spouses, but the present-biased spouses conceal money to counteract this strategy. Our study indicates the importance of external savings commitment devices in helping people protect money from their present-biased spouses.

## Introduction

Empirical evidence suggests that people do not save as much as they think they should [1, 2]. Several studies have identified present bias as a major reason for under-saving [3–5]. Present-biased individuals place a particularly high value on immediate consumption and often spend their earnings immediately without saving much for the future [6].

Individuals who understand their present bias should value savings commitment devices, such as retirement savings plans and savings plans with withdrawal restrictions [4, 7–9]. In developing countries, people have limited access to such savings commitment devices through formal financial institutions and, hence, often utilize informal financial arrangements, such as rotating savings and credit associations (ROSCAs) and deposit collectors for savings commitment devices [10–12]. Microcredit, a lending scheme for the poor with regular and frequent repayments, is also used as a commitment device when it is accessible [13].

In this study, we investigate whether married couples utilize their spouses as a savings commitment device to solve the problem of present bias. In many cultures, households are the primary units of consumption and savings, and marriage is often considered a financial contract between spouses [14–16]. Household income is often pooled between a husband and wife, and, generally, one spouse is in charge of daily consumption decisions [17]. By allowing a present-biased spouse to delegate the consumption decision to his/her partner who is not present biased, marriage can function as a savings commitment device.

Although existing studies have not investigated the possibility that marriage functions as a savings commitment device, marriage may serve this function for several potential reasons. First, spouses have strong incentives to help their partners solve the present bias problem, as a couple shares a household budget. Further, spouses can observe the actions and states of their partners quite well, mitigating the conflict between commitment and flexibility in designing the structure of a commitment device [18, 19]. However, spouses might dislike giving strong decision power to their partners [20] or might want to hide private income from their partners [17], especially when the two spouses differ in their time preferences [21]. Nevertheless, the effect of spouses’ present bias on the structure of household financial control and whether marriage can alleviate the individual present bias problem has not been previously studied.

This study investigates whether present-biased individuals use their spouses as savings commitment devices by analyzing experimental and survey data collected in Vietnam. Whereas individuals become less present-biased when they jointly make decisions with their spouses in experimental games, the analysis of the survey data shows that present-biased individuals turn over a smaller share of their earnings to their spouses, withholding larger amounts of income for themselves. These individuals also receive more money from their spouses’ incomes and are more likely to hold cash at their disposal. These results hold irrespective of whether couples jointly make non-present-biased decisions. Hence, married couples not only fail to utilize their spouses as savings commitment devices but also provide more money for present-biased individuals to spend, exacerbating the problem. We also find that couples whose joint decisions are not present-biased do tend to allocate smaller allowances to present-biased individuals; however, the latter conceal money to counteract this strategy. We find that wives with present-biased husbands are more likely to participate in ROSCAs, which is consistent with a previous study in Kenya finding that wives use ROSCAs to protect money from their husbands [10]. Our study indicates the importance of savings commitment institutions, such as ROSCAs and savings accounts, that function outside of households and, thus, enable spouses to protect their finances from present-biased partners.

## Survey and experimental design

### Selection of subjects and survey procedure

We conducted a survey and economic experiment in one urban commune in Can Tho City in Vietnam in May and June 2010. T. Tanaka obtained the approval from IRB at Arizona State University in 2010, which she was affiliated at that time. We collected the written form of consent from all the participants, inserted in the questionnaire. We conducted the survey in collaboration with a faculty member in Can Tho University in Vietnam, who completed all the necessary documentation to obtain the permits and approval to the research from the government, including the permissions and approvals for foreign researchers to conduct the survey in presence of the faculty member from Can Tho University. All the research and experiments were conducted under his presence. The data were analyzed anonymously.

The commune was selected for the Vietnam Household Living Standard Survey (VHLSS 2002), a nationally representative survey conducted in 2002. According to the VHLSS 2002, the commune is densely populated and is a relatively wealthy commune in the region with a higher mean income than the regional average. The main job categories in Commune A are trade, transportation, and services. These areas are located in the same commune and have similar characteristics. Thus, the areas are not very different in terms of culture or economics.

We chose parents of first and second graders living in the commune as our subjects because we expect most such individuals to be married and economically active and, thus, to have a strong incentives to save for future educational investments. These incentives should generate a demand for a savings commitment device if individuals are present biased. The commune includes six communities, Area 1 through Area 6. We first requested that the head of each Area contact the parents of first graders and invite both spouses to the study. There were 205 first graders living in the commune at the time of recruitment. We excluded from the subject pool 45 parents who were divorced or separated. Another 45 couples did not want to participate in the study. Therefore, we conducted the survey with the remaining 115 parents, resulting in a participation rate of 72%. Subsequently, we began to recruit parents of second graders in Areas 1, 2, and 3 until we had collected data from 150 couples (300 parents). Most subjects lived within five minutes of the commune office (by motorbike or bicycle). We exclude data from the first 16 couples, as a slightly different experimental design was used for these couples. On the first day of the survey, when these 16 couples were surveyed, we listed ten experimental questions and requested that the subjects answer each of them [22]. However, some subjects were confused about how to read the tables. From the second day forward, we showed subjects the questions one by one, which facilitated their understanding of our experimental games. The results are similar when we include the 16 couples from the first day of the survey in the following analyses. After dropping these 16 couples, we construct the final dataset of 134 couples.

When a couple arrived at the commune office, we conducted a household survey with both spouses to obtain data on household demographics, income, properties, and financial management within the household. After the household survey, we conducted separate experiments with both husbands and wives. We prepared private rooms for husbands on the second floor of the commune office building and for wives on the first floor. After running the experiment, we conducted an additional survey with the individual subjects (without their spouses) to gather data on the proportion of earnings turned over to spouses, the value of properties inherited from their parents, and participation in ROSCAs. We also tested each subject’s arithmetic skills and financial literacy by eight arithmetic problems and three financial literacy questions. The arithmetic questions consisted of addition, subtraction, multiplication, and division. The three financial literacy questions were as follows: (1) Suppose you had one million VND in a savings account and the interest rate was 2% per year. After five years, how much do you think you would have in the account if you left the money to grow: more than 1.02 million VND, exactly 1.02 million VND, or less than 1.02 million VND?(2) Imagine that the interest rate on your savings account was 1% per year and inflation was 2% per year. After one year, would you be able to buy more than, exactly the same as, or less than the amount you could buy today with the money in this account? (3) Suppose you had one million VND in a savings account and the interest rate was 5% per year. After ten years, how much do you think you would have in the account if you left the money to grow: more than 1.5 million VND, exactly 1.5 million VND, or less than 1.5 million VND?

Table 1 reports the summary statistics of the subjects. On average, our subjects are 37.5 years old and have eight years of education. The average monthly salaries for husbands and wives are 2.5 million VND (US$120.85) and 1.4 million VND (US$67.68), respectively, and they are statistically significantly different at the 1% level. Wives tend to be in charge of household budgets. Husbands turn over 71.9% of their income to their wives, whereas wives turn over 13% of their income to their husbands. Among husbands, 32.3% are responsible for holding cash within households, whereas the corresponding figure for wives is 86.6%. Husbands and wives receive 648,000 VND and 406,000 VND in monthly allowances, respectively. As for occupation, 43 and 31 of the 134 male subjects work for private enterprises and other households (including casual work), respectively, and 29 are self-employed. Among the female subjects, 34 work for other households (including casual work), 24 are not currently working, and 23 are self-employed. This pattern is consistent with that found in the VHLSS 2002.

**Table 1:** Summary statistics.

	Male	Female	Total	Difference between male and female
Age	38.56 (6.530)	36.40 (6.782)	37.48 (6.733)	2.164*** (0.813)
Education	8.104 (3.419)	7.910 (3.626)	8.007 (3.519)	0.194 (0.431)
Percent of correct answers to the arithmetic problems	0.611 (0.316)	0.582 (0.321)	0.597 (0.318)	0.029 (0.039)
Percent of correct answers to the financial literacy questions	0.607 (0.344)	0.485 (0.331)	0.546 (0.343)	0.122*** (0.041)
Own monthly income: million VND	2.528 (1.819)	1.442 (1.515)	1.985 (1.757)	1.086*** (0.204)
Value of own assets: million VND	280.0 (495.6)	222.8 (602.8)	251.4 (551.5)	57.260 (67.415)
Value of own inherited assets: million VND	128.5 (380.6)	97.48 (245.5)	113.0 (320.0)	31.022 (39.121)
Percent of subject’s salary given to his/her spouse	0.719 (0.318)	0.130 (0.297)	0.446 (0.426)	0.590*** (0.040)
Keep cash in the household (yes = 1, no = 0)	0.321 (0.469)	0.866 (0.342)	0.593 (0.492)	-0.545*** (0.050)
Amount of allowance per month: thousand VND	648.1 (553.2)	406.8 (411.1)	527.5 (501.2)	241.306*** (59.539)
Hidden disposable money	-10.15 (547.7)	217.6 (742.7)	103.7 (661.2)	-227.724*** (79.719)
ROSCA participation	0.0821 (0.276)	0.127 (0.334)	0.104 (0.306)	-0.045 (0.037)
Any savings (yes = 1, no = 0)	0.612 (0.489)	0.612 (0.489)	0.612 (0.488)	
Present bias (yes = 1, no = 0)	0.336 (0.474)	0.403 (0.492)	0.369 (0.484)	-0.067 (0.059)
Joint decision present bias (yes = 1, no = 0)	0.231 (0.423)	0.231 (0.423)	0.231 (0.422)	
Discount factor	0.935 (0.0478)	0.943 (0.0461)	0.939 (0.0470)	-0.008 (0.006)
Joint decision discount factor	0.935 (0.0474)	0.935 (0.0474)	0.935 (0.0473)	
Sophisticated present bias (yes = 1, no = 0)	0.291 (0.456)	0.336 (0.474)	0.313 (0.465)	-0.045 (0.057)

Note: Mean values are reported with standard errors in parentheses. Asterisks indicate statistically significant differences between husbands and wives:

* *p* < .10,

** *p* < .05, and

*** *p* < .01.

Savings and joint decision variables are common within a couple, so we do not report the differences in these variables.

### Experimental games

The experiment included several versions of time-discounting games (TDGs). In Individual TDG I, subjects were asked to choose to receive either 100,000 VND (US$4.83) today or a larger amount of money in three days in each of ten questions [22]. The delayed payments varied from 100,000 VND to 145,000 VND. In Individual TDG II, subjects chose either 100,000 VND in three weeks or a larger amount of money in three weeks and three days. As argued in the next section, these two games identify present-biased individuals [7, 8]. To check whether subjects were aware of their own present bias problems, we gave them an opportunity to change their decisions for Individual TDG II three weeks after the experiment. Sophisticated subjects who are aware of the present bias problem should voluntarily commit to their initial decisions.

In these games, subjects made decisions individually in separate rooms. Then, we conducted similar games that only differed in that subjects and their spouses could speak to each other over the experimenter’s phone and make joint decisions (called “Joint TDG I” and “Joint TDG II,” respectively).

The subjects also participated in other games, including risk games, in which subjects chose one risky option out of six options available to them [23] using individual and joint decision-making. Only the choices in the TDGs are used for the research question in this study, and, thus, we only focus on the TDGs.

At the end of the experiment, one game was selected for payment. Subjects threw a die to determine the game used for payment. If either Joint TDG I or II was selected for payment, the joint decision made by the couple was used for the individual subject’s payment. Subjects were also paid 100,000 VND (US$4.83) as a show-up fee, which is equivalent to 4% and 7% of monthly income for husbands and wives, respectively. If a delayed payment was selected as the payment, subjects received only the show-up fee on the day of the experiment. To minimize the possibility that the unreliability of future rewards would affect subjects’ decisions in the TDGs [24], we entrusted the envelopes containing the experimental rewards for these subjects to the chief of the commune office and provided a special folder for these envelopes. We checked the folder on every visit to the commune office to ensure that the payments were secure and that no envelope was missing. Although the transaction costs for receiving future rewards could be a concern [24], in practice, all subjects visited the office to pick up their delayed payments, and, hence, transaction costs barely affected subjects’ decisions in our TDGs.

## Results

### Time preferences

We elicited time preferences from the subjects’ choices in Individual TDGs I and II. The choices in Individual TDG I identify time preferences over today and three days later, and the choices in Individual TDG II identify time preferences over three weeks from today and three weeks from today plus three days later. If subjects are time consistent, the choices should be the same for Individual TDGs I and II. However, the Wilcoxon signed-rank test rejects this null hypothesis (*p* < .0001). We define those subjects whose choices were more impatient in Individual TDG I than in Individual TDG II as present biased (PB).

The distribution of present bias is shown in Table 2. We mainly focus on the binomial indicator for present bias. We could compute the degree of present-biasedness, *β*, from the subjects’ choices; however, the elicited value is relatively high compared to that found by other studies [25]. This difference might be due to the limited variation in the amount of experimental rewards, with which the minimum value of *β* possible in our experimental design is 0.69.

**Table 2:** Elicited time preferences.

Husband	Wife		
	Not present-biased	Present-biased	Total
Not present-biased	38.8 (PB in joint decision: 2.2)	27.6 (PB in joint decision: 6.7)	66.4 (PB in joint decision: 9.0)
Present-biased	20.9 (PB in joint decision: 6.7)	12.7 (PB in joint decision: 7.5)	33.6 (PB in joint decision: 14.2)
Total	59.7 (PB in joint decision: 9.0)	40.3 (PB in joint decision: 14.2)	100.0 (PB in joint decision: 23.1)

The table reports the fraction (%) of the total number of subjects belonging to each cell. The fractions (%) of couples who exhibit present bias in their joint decisions are in parentheses.

We find that 34% of husbands and 40% of wives are PB. This difference is not statistically significant (*p* = 0.2659). Although 38.8% of couples consist of two non-PB individuals and 12.7% consist of two PB individuals, nearly half of the couples consist of one PB and one non-PB individual. In the latter type of couple, the PB individual can utilize his/her spouse as a savings commitment device. However, if the time discount factor, *δ*, differs substantially within a couple, asking his/her spouse to manage the budget will be suboptimal even for the PB individual. Hence, in this analysis, we control for an individual’s own discount factor and the spouse’s discount factor, which can be elicited from the choices in Individual TDG II. The value of *δ* turns out to be larger for wives than for husbands (0.935 and 0.943, respectively), although the difference is insignificant (*p* = 0.137).

Using the results from Joint TDGs I and II, we measure the joint decision present bias and the joint decision discount factor. The fraction of couples whose joint decision is PB is 23.1%, which is much smaller than in the case of individual decision-making (36.9%). The fractions of couples who exhibit present bias in their joint decisions are reported in parentheses in Table 2. Couples with no individually PB members rarely exhibit present bias in their joint decisions. Only 28% ((6.7+6.7)/(27.6+20.9)) of couples with one PB member exhibit present bias in their joint decisions. Even when both members of a couple are PB, they are not PB in their joint decision 41% ((12.7-5.2)/12.7) of the time. This result is consistent with previous studies that show that group decision-making mitigates the present bias problem [26–29]. This result implies that joint decision-making within marriages could also alleviate individual present bias.

### Intrahousehold income transfer

We now investigate whether PB individuals utilize their spouses as savings commitment devices by analyzing actual household financial decision-making. First, we examine the share of earnings turned over to spouses. This variable is calculated as the earnings turned over to an individual’s spouse divided by the sum of earnings kept by that individual and those turned over to his/her spouse. We exclude any earnings turned over to other persons, such as parents, because they do not relate to the present bias problems within a couple. Note that not all subjects have earnings to turn over (e.g., housewives), which reduces our sample size to 127 husbands and 110 wives (out of 134 each).

S1 Fig shows a histogram of the share of earnings turned over to spouses. Whereas over half of husbands entrust more than 80% of their earnings to their wives, around 80% of wives keep all of their own earnings. This finding reflects a common aspect of Asian cultures in which wives tend to be in charge of household budgeting [17]. The figure also shows that the proportion of individuals who keep all of their earnings is larger in the PB samples. To control for other variables that affect the decision to turn one’s earnings over to one’s spouse and are correlated with the present bias, we conduct regression analyses. As the share of earnings turned over ranges from zero to one (i.e. the corner solution outcome [30]) and substantial proportions of observations have values of zero or one (S1 Fig), we use the following two-limit Tobit model:

$$\begin{array}{ccc} y_{i}^{*} & = & w_{i}\theta +x_{i}\gamma +u_{i}\;u_{i}|w_{i},x_{i}∼N\left(0,\sigma^{2}\right)\\ y_{i} & = & \left\{ \begin{array}{cc} 0 & \;\text{if}\;y_{i}^{*}\leq 0\\ y_{i}^{*} & \;\text{if}\;0<y_{i}^{*}<1\\ 1 & \;\text{if}\;y_{i}^{*}\geq 1 \end{array},\right. \end{array}$$

where *y*_*i*_ is the share of earnings of individual *i* turned over to his/her spouse, $y_{i}^{*}$ is the latent variable, *w*_*i*_ is a row vector of present bias variables, *x*_*i*_ is a row vector of other observable controls that affect intrahousehold decision-making (i.e., gender; the time discount factor; differences in age, education, earnings, own assets, own inherited assets, arithmetic score, and financial literacy score; and years of marriage, which captures trust between spouses), and the column vectors, *θ* and *γ*, are parameters to be estimated. Standard errors are clustered at the couple level to consider intra-couple correlations. We use two specifications for the vector of present bias variables, *w*_*i*_. The first specification includes indicator variables for being PB and for one’s spouse being PB. In the second specification, we consider the possibility that a PB individual may not have an incentive to turn over his/her earnings if his/her spouse is also PB by including indicator variables for (i) a PB individual with a non-PB spouse (“PB & Spouse is not PB”), (ii) a non-PB individual with a PB spouse (“Not PB & Spouse is PB”), and (iii) a PB individual with a PB spouse (“PB & Spouse is PB”). Couples in which neither spouse is PB is the reference category. Given that couples were less likely to exhibit present bias in the joint decision case, we also run the regressions including the joint decision present bias. As shown in Table 2, the proportion of couples who were PB in the joint decision case is not large. Thus, we only include the joint decision present bias in the first specification so that our estimates do not rely on a small number of observations.

We also estimate the models that include a variable for sophistication, but one needs caution in interpretation, as the number of unsophisticated PB subjects is quite small; only 15 subjects exhibit unsophisticated PB, and, of these, only seven have non-PB spouses. In addition, naive individuals do not necessarily accept the option to change their decision (e.g., they may believe that they would make the same choice in three weeks and feel it cumbersome to decide again and, thus, reject the offer), and, hence, the set of subjects classified as sophisticated PB might include some naive individuals.

The regression results are presented in Table 3. If marriage functions as a commitment device, we should observe PB individuals turning over earnings to their spouses. Column (1), however, shows contrary results. PB individuals turn over a smaller share of their earnings, and individuals turn over more earnings when their spouses are PB. This result implies that PB individuals tend to hold more household resources. Marriage not only fails to function as a commitment device, but it also exacerbates the problem by providing additional resources for PB individuals to consume. Considering the possibility that a PB individual may have little incentives to turn over earnings if his/her spouse is also PB does not change the results. As shown in Column (8), PB individuals turn over less money when their spouses are not PB, and non-PB individuals turn over more money when their spouses are PB.

**Table 3:** The share of earnings turned over to spouses.

First specification							
	(1) All	(2) All	(3) Husband	(4) Wife	(5) All	(6) Husband	(7) Wife
Present-biased (PB)	-0.264* (0.140)	-0.257 (0.160)	-0.191 (0.126)	-0.561 (0.475)	-0.212 (0.277)	-0.140 (0.232)	-0.038 (0.939)
Spouse is PB	0.424*** (0.144)	0.423*** (0.150)	0.277** (0.138)	1.413*** (0.521)	0.424*** (0.144)	0.277** (0.138)	1.389*** (0.524)
PB & Joint decision non-PB		-0.011 (0.181)					
Sophisticated PB					-0.060 (0.260)	-0.057 (0.229)	-0.600 (0.991)
Control	Yes	Yes	Yes	Yes	Yes	Yes	Yes
Observations	237	237	127	110	237	127	110
Second specification							
	(8) All	(9) Husband	(10) Wife	(11) All	(12) Husband	(13) Wife	
PB & Spouse is not PB	-0.337*** (0.162)	-0.253 (0.161)	-0.612 (0.607)	-0.396 (0.315)	-0.187 (0.305)	-7.704*** (0.187)	
Not PB & Spouse is PB	0.359** (0.162)	0.224 (0.159)	1.369** (0.618)	0.358** (0.161)	0.226 (0.160)	1.376*** (0.103)	
PB & Spouse is PB	0.202 (0.188)	0.119 (0.178)	0.872 (0.727)	0.200 (0.187)	0.122 (0.178)	0.879*** (0.095)	
PB & Spouse is not PB × sophisticated PB				0.065 (0.308)	-0.073 (0.303)	7.141*** (0.187)	
Control	Yes	Yes	Yes	Yes	Yes	Yes	
Observations	237	127	110	237	127	110	

This table reports the estimated average partial effects of the Tobit model. The control variables include a female dummy (if applicable) and standardized differences in income, assets, age, education, arithmetic score, and financial literacy. Standard errors clustered by couple are in parentheses. Asterisks indicate statistical significance:

* *p* < .10,

** *p* < .05, and

*** *p* < .01.

We also find that individuals turn over more earnings when they are more patient and when their spouses are less patient. Wives turn over significantly fewer earnings, reflecting the social context in which wives are usually in charge of household budgets. To check for possible misspecifications of the Tobit model, we also estimate corresponding alternative-ordered probit models [30], but we still find similar results.

We have seen that couples are less likely to be PB in Joint TDGs I and II. However, this de-biasing effect of joint decision-making does not seem to alleviate the present bias problem. Adding an indicator variable for being individually but not jointly PB (“PB & Joint decision non-PB”) has little effect on the results (Column (2)). The only difference is that the effect of own present bias is insignificant owing to the larger standard error caused by the collinearity between these two variables. The coefficient on the joint decision variable is insignificant and close to zero.

In Columns (3)-(4) and (9)-(10), we split the sample into husbands and wives, as the behavior could differ for husbands and wives owing to intrahousehold bargaining power and social norms. Although the coefficients on the indicators for one’s own PB and for a PB individual with a non-PB spouse are insignificant, likely owing to the smaller sample size, the coefficient on the spouse’s PB variable is still significant for both husbands and wives. Strikingly, we observe a larger effect of a PB spouse in the sample of wives, indicating that women suffer more from their PB husbands.

One possible reason that marriage does not function as a commitment device is that spouses are not aware of their own present bias problem and, thus, have no demand for commitment. To examine this possibility, we directly elicit the demand for commitment. After Individual TDG II, in which the subjects made decisions between three weeks from today and three weeks from today plus three days later, we offered them the option to change their decision in three weeks. Individuals who are aware of their present bias problem should voluntarily commit to their initial decision [24, 31] and, thus, reject this offer. Those who are not aware of their present bias problem should accept this offer for the sake of flexibility. We define subjects who exhibit PB but reject this offer as sophisticated PB subjects. As reported in Table 1, 31.3% of the subjects are sophisticated PB, whereas the total fraction of PB subjects is 36.9%. This result is consistent with our presumption that most PB individuals are aware of their problem.

The estimation results reported in Columns (5)-(7) and (11)-(13) in Table 3 do not indicate that sophisticated PB individuals utilize their spouses as commitment devices. The PB variable no longer has a significant effect owing to the collinearity between the PB variable and the sophistication variable; however, the linear combination of the PB variable and the sophistication variable still has a negative effect in Columns (5)-(7). The coefficient on the indicator for a sophisticated PB individual with a non-PB spouse (“PB & Spouse is not PB× sophisticated PB”) is positively significant and large for wives (Column (13)). However, the large negative coefficient on the indicator for a PB individual with a non-PB spouse (“PB & Spouse is not PB”) implies that the linear combination is negative and insignificant. Thus, sophisticated PB wives do not turn over more earnings to their non-PB husbands than wives free from present bias do. We find few changes in the coefficients on the indicators for non-PB individuals with PB spouses. We also examine whether sophisticated PB individuals whose joint decisions with their spouses are not PB behave differently, but we find no significant differences. Overall, including the sophistication variable does not change the result that individuals turn over more earnings if their spouses are PB.

Other potential reasons that PB individuals may not turn over more earnings to their non-PB spouses include a lack of trust or differences in preferences for intratemporal consumption between spouses. However, this rationale cannot explain why individuals turn over more earnings when their spouses are PB. One may also think that transfers to PB spouses are driven by pressure from PB spouses. In that case, however, individuals with greater intrahousehold bargaining power would transfer less money to their PB spouses. However, when we include the interactions between the PB variables and variables relating to intrahousehold bargaining power, such as differences in the values of own assets, education, and age, we find no evidence that individuals with greater bargaining power turn over fewer earnings to their PB spouses, as reported in S1 Table. Rather, PB individuals with more assets turn over a larger share of their earnings to their spouses, and husbands with PB wives turn over more earnings when the age difference is larger. Perhaps, spouses find it difficult to reject requests from their PB partners simply because they want to be nice to their spouses, although we have no direct evidence supporting this argument.

Another potential concern is that partner matching may be affected by time preferences. Table 2, however, shows no matching patterns, and Pearson’s chi-squared test cannot reject the null hypothesis that the husband’s and wife’s PB indicators are independent (*p* = 0.50). Nevertheless, couples with different time preferences may differ from couples with similar time preferences in that they may have married because they believed that they could manage the present bias problem. In that case, however, we would likely find couples using their spouses as savings commitment devices, whereas we instead find the opposite. Thus, the possibility of endogenous matching does not explain our results.

One can also imagine a different matching pattern in which a PB individual who has some advantage in the marriage market marries a non-PB partner who is willing to turn over his/her earnings. However, in this case, our conclusion that PB individuals fail to use their spouses as savings commitment devices and are provided more resources to spend still holds. Omitted variables are another possible concern. However, among the factors that affect the share of earnings turned over to spouses, bargaining power is already controlled for by differences in income, assets, age, education, arithmetic score, and financial literacy. Another factor that may influence the share of earnings turned over is generosity. If individuals with PB spouses are more generous, then our estimated coefficients on the indicator for a PB spouse capture the effect of generosity. In this case, however, the result is that PB individuals fail to use their spouses as savings commitment devices and receive more resources, again leaving the conclusion unchanged. Thus, considering the possibility of omitted variables does not affect our conclusion.

### Financial manager

Next, we investigate the pattern of intrahousehold financial management. We ask our subjects who keeps cash in their households, allowing for multiple answers, and we identify individuals that keep cash as financial managers. As shown in Table 1, wives are much more likely to be financial managers in the household (86.6% of wives, 32.3% of husbands), as is often observed in Asian societies.

Table 4 reports the estimated average partial effects of the probit model in which the dependent variable is an indicator for being the financial manager. As the dependent variable is the indicator for keeping cash (not delegating the role of financial manager), we expect the coefficients to have the opposite signs to those in Table 3, in which the outcome variable is the share of earnings turned over to one’s spouse. We also report the estimated coefficients for the linear probability model in S2 Table.

**Table 4:** Subjects who are financial managers in their households.

First specification							
	(1) All	(2) All	(3) Husband	(4) Wife	(5) All	(6) Husband	(7) Wife
Present-biased (PB)	0.098* (0.054)	0.037 (0.067)	0.238** (0.105)	-0.009 (0.059)	0.326** (0.130)	0.436** (0.200)	0.749*** (0.149)
Spouse is PB	-0.032 (0.061)	-0.021 (0.061)	-0.061 (0.095)	-0.030 (0.076)	-0.030 (0.060)	-0.056 (0.095)	-0.032 (0.075)
PB & Joint decision non-PB		0.096 (0.079)					
Sophisticated PB					-0.250* (0.130)	-0.215 (0.193)	-0.770*** (0.151)
Control	Yes	Yes	Yes	Yes	Yes	Yes	Yes
Obs.	268	268	134	134	268	134	134
First specification							
	(8) All	(9) Husband	(10) Wife	(11) All	(12) Husband	(13) Wife	
PB & Spouse is not PB	0.086 (0.062)	0.262** (0.124)	-0.043 (0.071)	0.398** (0.171)	0.530** (0.239)	0.741*** (0.148)	
Not PB & Spouse is PB	-0.044 (0.062)	-0.038 (0.109)	-0.071 (0.089)	-0.035 (0.061)	-0.024 (0.109)	-0.068 (0.087)	
PB & Spouse is PB	0.076 (0.068)	0.164 (0.144)	-0.013 (0.104)	0.086 (0.067)	0.179 (0.145)	-0.008 (0.102)	
PB & Spouse is not PB × sophisticated PB				-0.335* (0.176)	-0.292 (0.235)	-0.794*** (0.155)	
Control	Yes	Yes	Yes	Yes	Yes	Yes	
Obs.	268	134	134	268	134	134	

This table reports the estimated average partial effects of the probit model. We include the same control variables as in Table 3. Standard errors clustered by couple are in parentheses. Asterisks indicate statistical significance:

* *p* < .10,

** *p* < .05, and

*** *p* < .01.

If marriage functions as a commitment device, then PB individuals should delegate the financial management role to their non-PB spouses. However, we do not find any evidence supporting this story. If anything, PB individuals, especially husbands or PB husbands whose wives are not PB, are more likely to keep cash, as shown in Columns (1)-(4) and (8)-(10). These results are consistent with those for intrahousehold income transfers, which show that PB individuals tend to hold more household resources.

When we include the sophistication variable (Columns (5)-(7) and (11)-(13)), we find that sophisticated PB individuals behave similarly to non-PB individuals, whereas naive PB individuals are more likely to keep cash than non-PB individuals are. Although sophistication alleviates the problem, even sophisticated individuals do not actively utilize marriage as a commitment device because they are not less likely to keep cash. As noted before, one need caution in interpreting the results because the number of unsophisticated PB subjects is quite small and hence the identification of the parameters depends on a small number of observations. This issue might explain the large magnitudes of the average partial effects in the probit model, especially for wives. When we use the linear probability model (S2 Table), the magnitudes of the coefficients are smaller, and the coefficients are insignificant for wive.

Unlike in the analysis of the share of earnings turned over, the spouse’s PB variable has no significant effect because both spouses can keep cash without asking their partners, rendering the effect of the spouse’s present bias negligible. These patterns are observed in samples of both husbands and wives. We also run a regression including the interaction terms with bargaining power measures; however, these terms do not appear to have a significant effect. The results are available upon request.

### Pocket money

We now turn to the reallocation of pooled resources. Specifically, we examine the amounts of money that couples allocate to each other as monthly allowances. Although PB individuals turn over smaller portions of their earnings, couples can manage the present bias problem by giving smaller allowances to PB individuals. Relative to the share of income turned over to spouses, the allowance allocated to each spouse is more likely to be under the control of the financial manager or to be a couple’s joint decision. Couples that make joint decisions and are time consistent may be able to alleviate the problem of individually PB spouses by reducing these spouses’ monthly allowances. To explore this possibility, we asked couples in the household survey how much money they received as allowances in the previous month. As reported in Table 1, allowances are significantly larger for husbands than for wives (648,000 VND for husbands and 406,800 VND for wives).

The upper panel of Table 5 reports the regression results for monthly allowances. We use the same covariates as before and also control for the size of household income because the income level affects the allowance amount. The results in Column (1) suggest that PB individuals actually receive smaller allowances. The results in Column (2) suggest that this effect is driven by couples whose joint decisions are not PB; such couples allocate smaller allowances to PB individuals, whereas couples whose joint decisions are PB fail to reduce the monthly allowances to PB individuals.

**Table 5:** Monthly allowances and hidden disposable money.

	(1) All	(2) All	(3) Husband	(4) Wife	(5) Husband	(6) Wife
**Monthly allowance**						
Present-biased (PB)	-164.5** (66.4)	-44.7 (79.3)	-263.7** (125.8)	-58.7 (73.7)	-248.6* (143.0)	102.4 (113.6)
Spouse is PB	-98.5 (72.8)	-120.9 (74.5)	18.6 (86.1)	-253.7** (124.2)	15.9 (89.6)	-289.9* (126.2)
PB & Joint decision non-PB		-182.7** (82.0)			-26.2 (128.6)	-230.4** (112.9)
Control	Yes	Yes	Yes	Yes	Yes	Yes
Obs.	268	268	134	134	134	134
**Hidden disposable money**						
Present-biased (PB)	192.6* (101.2)	72.9 (104.5)	120.4 (106.5)	220.6 (149.6)	84.8 (141.5)	34.3 (159.4)
Spouse is PB	-98.9 (108.0)	-76.5 (102.2)	-9.6 (70.2)	-184.5 (250.1)	-3.1 (73.8)	-142.6 (239.1)
PB & Joint decision non-PB		182.6 (123.6)			61.5 (145.6)	266.5 (204.3)
Control	Yes	Yes	Yes	Yes	Yes	Yes
Obs.	268	268	134	134	134	134

This table reports the estimated coefficients of an ordinary least squares regression. We include the same control variables as in Table 3 as well as a variable for household income level. Standard errors clustered by couple are in parentheses. Asterisks indicate statistical significance:

* *p* < .10,

** *p* < .05, and

*** *p* < .01.

We find more subtle results when separating the sample into husbands and wives. The results in Columns (3) and (4) suggest that when husbands are PB, *both* husbands’ and wives’ monthly allowances are 253,700 VND lower, which is more than 50% of the average allowance for wives. Whether or not wives are PB does not affect their allowances. The results in Columns (5) and (6) suggest that although the present bias in the joint decision does not affect the allowances of PB husbands, it does affect those of PB wives. One concern may be that the negative coefficients on the PB variable arise because PB individuals turn over fewer earnings to their spouses or to the household budget. However, including the share of earnings turned over to spouses as a regressor slightly increases the magnitudes of the coefficients on PB variables, as reported in the upper panel of S3 Table. Clearly, this variable is affected by time preferences and, hence, is a bad control [32], but it captures the omitted variables related to the smaller household budget caused by having less pooled income. Thus, our finding that a PB individual receives a smaller allowance is not driven by the smaller amount of earnings turned over.

Although we find some evidence that couples try to alleviate the present bias problem by allocating smaller allowances to PB individuals, it is still possible for these PB individuals to counteract this strategy by hiding and keeping part of their incomes. To examine this possibility, we asked subjects individually about the amount of money they could spend without their spouse’s agreement. From this data, we calculate the hidden disposable money as the difference between a subject’s monthly allowance (from the household survey) and the amount of money that the subject can spend without his/her spouse’s agreement. The lower panel of Table 5 reports the regression results. Column (1) shows that the coefficient on own PB is significantly positive and that its magnitude is comparable to that of the monthly allowance regression (Column (1) in the upper panel). This result suggests that although households allocate smaller allowances to PB individuals, these individuals hide an equivalent amount of money to maintain enough money for spending. This is consistent with recent literature showing that hiding income from his/her spouse is often observed in the presence of asymmetric information [33].

We find a similar pattern in Column (2), which includes an indicator variable for being individually but not jointly PB. Again, although households try to alleviate the present bias problem by allocating less money to PB individuals, these individuals hide their incomes and counteract these efforts. When we split the sample into husbands and wives, the significance vanishes, probably owing to the smaller sample size; however, we still find that PB individuals hide more money to counteract the allowance reduction. When the reduction in allowance is due to the husband’s present bias, however, wives do not (or possibly cannot) increase the amount of hidden money.

These results do not change if we control for the share of earnings turned over to spouses (S3 Table in SI). Note that the amount of hidden disposable money (say, *H*) is the difference between the amount of money a subject can spend without his/her spouse’s agreement (say, *M*) and his/her monthly allowance (say, *A*), that is, *H* = *M* − *A*. If *M* is constant across individuals, then any variables positively correlating with *A* correlate negatively with *H* by construction. S2 Fig in SI indicates that the value of *M* is not constant and is distributed as sparsely as the monthly allowance is. Furthermore, the correlation between *H* and *A* is positive, not negative, with a correlation coefficient of 0.52. When we regress the value of *M* on the same set of control variables, the coefficient on the indicator of own PB is insignificant, indicating that the amount of money that a subject can spend without his/her spouse’s agreement is not influenced by the subject’s PB status.

### ROSCAs

Previous literature argues that ROSCAs function as commitment mechanisms to protect money from one’s own present bias problem and spousal pressure for money [10]. In this subsection, we examine whether participation in ROSCAs correlates with own and spouse’s present bias. Note that only 28 out of the 268 subjects report participating in ROSCAs, and 12 of these 28 subjects are in the same households. In these cases, it is not clear whether both husbands and wives have access to the money in ROSCAs or if only one of them is part of a ROSCA but both report using ROSCAs. Hence, we need to be cautious in interpreting the results. Table 6 reports the estimation results of the probit models and shows that couples consisting of one PB spouse and one non-PB spouse are more likely to use ROSCAs (Column (3)). Columns (6)-(7) indicate that non-PB wives are more likely to participate in ROSCAs when their spouses are PB, although the results are only significant at the 10% level. Note that in the previous analyses, we found that non-PB wives with PB husbands turn over a substantially larger share of their earnings and receive less income from their husbands. Wives with PB husbands also receive smaller allowances and, although the result is statistically insignificant, less disposable money. Given these results, it is likely that these wives utilize ROSCAs to protect their income from their PB husbands. Note that when we include own PB and spouse’s PB, the coefficients are insignificant (Columns (1)-(2) and (4)-(5)). Hence, these results must be interpreted with caution. We also report the estimated coefficients of the linear probability model in S4 Table. These results are similar to those in Table 6, although the results for wives are no longer significant.

**Table 6:** Participation in ROSCAs.

	(1) All	(2) All	(3) All	(4) Husband	(5) Wife	(6) Husband	(7) Wife
Present-biased (PB)	0.025 (0.046)	-0.020 (0.067)		0.032 (0.074)	0.016 (0.065)		
Spouse is PB	0.035 (0.053)	0.042 (0.053)		0.032 (0.070)	0.070 (0.088)		
PB & Joint decision non-PB		0.066 (0.065)					
PB & Spouse is not PB			0.112* (0.058)			0.137 (0.090)	0.093 (0.071)
Not PB & Spouse is PB			0.121* (0.066)			0.116 (0.087)	0.160* (0.096)
PB & Spouse is PB			-0.023 (0.094)			0.000 (.)	0.034 (0.123)
Control	Yes	Yes	Yes	Yes	Yes	Yes	Yes
Observations	268	268	268	134	134	117	134

The table reports the estimated average partial effects of the probit model. The same control variables as in Table 3 are included. For the sample of husbands, there are no observations for which both spouses are PB and the husband joined a ROSCA; therefore, these observations are dropped from the estimation, leaving us with 117 observations. Standard errors clustered by couple are in parentheses. Asterisks indicate statistical significance:

* *p* < .10,

** *p* < .05, and

*** *p* < .01.

We also investigate whether the actual accumulation of savings and assets is affected by the PB status of the spouses, but we do not find any significant effects (S5 Table). Given the measurement error and large variation in these variables, it is difficult to detect any impacts with a small sample size.

### Robustness

We found that 37% of subjects always choose to receive 100,000 VND sooner and never choose to receive a larger amount of money later both in Individual TDGs I and II. In the above analyses, we assumed that these subjects were not PB. S6 Table in SI reports the results when we drop these observations. Most of the estimated coefficients remain stable, although the estimates become less precise owing to the smaller sample size. Columns (1)-(3) in the upper panel of S6 Table report the estimation results for the share of earnings turned over to spouses. Our main finding still holds; PB individuals turn over fewer earnings to non-PB spouses, and non-PB individuals turn over more earnings to PB spouses. When we split the sample into husbands and wives, the significance vanishes, but the coefficients are still stable, and those for wives are much higher than those for husbands. Columns (4)-(5) report the estimation results for the financial manager role and show a similar pattern; although unsophisticated PB individuals are more likely to keep cash than non-PB individuals are, sophisticated PB individuals do not actively use their spouses as commitment devices. The middle panel of S6 Table reports the estimation results for monthly allowances (Columns (1)-(2)) and hidden disposable money (Columns (3)-(4)), showing similar results to those of the main analyses. PB individuals receive smaller monthly allowances, driven by those PB individuals who are not PB in their joint decisions. However, these individuals hide a corresponding amount of money to spend without their spouse’s agreement. Although these households try to alleviate the present bias problem by allocating less money to PB individuals, these individuals undo this strategy by hiding their income. Column (5) reports the estimation results for ROSCA participation using the full sample, and Column (6) reports those using only the sample of wives. The magnitudes of the coefficients are again similar.

In the lower panel of S6 Table, we drop those individuals that participate in ROSCAs. If PB individuals have already utilized an external commitment device, such as a ROSCA, they need not use their spouses as a commitment device, which might explain why they did not turn over more earnings to their spouses. However, excluding these observations, we still find similar results, which confirm that marriage fails in functioning as a commitment device.

## Conclusion

We examine whether marriage works as a savings commitment device. Although our experimental results suggest the possibility that joint decision-making may alleviate the present bias problem, actual household resource allocations exhibit the opposite pattern. PB individuals turn over fewer earnings to their spouses and receive more from their spouses. PB individuals are more likely to keep cash within households. These patterns hold irrespective of whether or not a couple’s joint decision exhibits present bias. Our results suggest that marriage not only fails to function as a savings commitment device but also exacerbates the present bias problem by providing more resources for PB individuals to consume. Households in which the joint decision is not PB actually allocate smaller monthly allowances to PB spouses; however, these individuals undo this strategy by concealing money. We also find that women suffer more from their PB husbands and that they are more likely to use ROSCAs.

Given that households exacerbate the present bias problem, the role of commitment devices outside of households is important. This finding may explain why previous studies find that married women tend to use ROSCAs. Our study cannot pin down the mechanisms by which couples allocate more resources to PB spouses. One potential factor may be the affection and acceptance that form the basis of marriage, which could result in the Samaritan’s dilemma. In other words, individuals consume more because they know their spouses will help them in need. Whereas recent studies focus on asymmetric information as the source of inefficiency in intra-household decision-making [17, 33, 34], the interaction between present bias and other-regarding preferences in intra-household decision-making may be another source of inefficiency. Another potential factor may be the implicit marriage contract in which a PB individual who has some advantage in the marriage market marries a non-PB partner with condition that the latter turns over his/her earnings. These analyses are left for future work.

## Supporting information

S1 Fig The percent of the subject’s salary turned over to his/her spouse. Histograms of the share of earnings turned over to spouses are depicted for husbands and wives, and for non present-biased individuals and present-biased individuals.
(PDF)

S2 Fig Kernel density estimates of the monthly allowance and monthly disposable spending without the spouse’s agreement. Estimated Kernel density function of the monthly allowance and monthly hidden disposal money are depicted.
(PDF)

S1 Table The share of earnings turned over to spouses: Including interaction terms for present bias and bargaining power. This table reports the estimated average partial effects of the Tobit model. The control variables include a female dummy (if applicable) and standardized differences in income, assets, age, education, arithmetic score, and financial literacy.
(PDF)

S2 Table Subjects who are financial managers in their households: Linear probability model. This table reports the estimated coefficients of the linear probability model. The control variables include a female dummy (if applicable) and standardized differences in income, assets, age, education, arithmetic score, and financial literacy.
(PDF)

S3 Table Allowance: Including the share of earnings turned over to the spouse. This table reports the estimated coefficients of an ordinary least squares regression. The control variables include a female dummy (if applicable) and standardized differences in income, assets, age, education, arithmetic score, and financial literacy.
(PDF)

S4 Table Participation in ROSCAs: Linear probability model. This table reports the estimated coefficients of the linear probability model. The same control variables as those used in Table 3 are included.
(PDF)

S5 Table Savings and assets. This table reports estimated average partial effects of the probit model (savings) and an ordinary least squares (assets) regression. The same control variables as in Table 3 are included. The sample of husbands includes no observations in which both spouses are PB and the husband joined a ROSCA; therefore, these observations are dropped from the estimation, leaving us with 117 observations.
(PDF)

S6 Table Robustness. This table reports the result of the robustness check in which the subjects who never shifted or the subjects who joined ROSCAs were dropped from the estimation.
(PDF)

S1 File Replication package. This zip file contains the STATA .do file and .dta file used to replicate the results of the manuscript.
(ZIP)
