# Supplementary material for: Does marriage work as a savings commitment device? Experimental evidence from Vietnam
Source: PLoS One. 2019 Jun 19;14(6):e0217646. doi: 10.1371/journal.pone.0217646 (PMC6583950; doi:10.1371/journal.pone.0217646)
Supplement: S1 Fig — Histograms of the share of earnings turned over to spouses are depicted for husbands and wives, and for non present-biased individuals and present-biased individuals. (PDF) [file pone.0217646.s001.pdf]

Supporting Information

S1 Fig. The percent of salary the subject turns over to his/her spouse

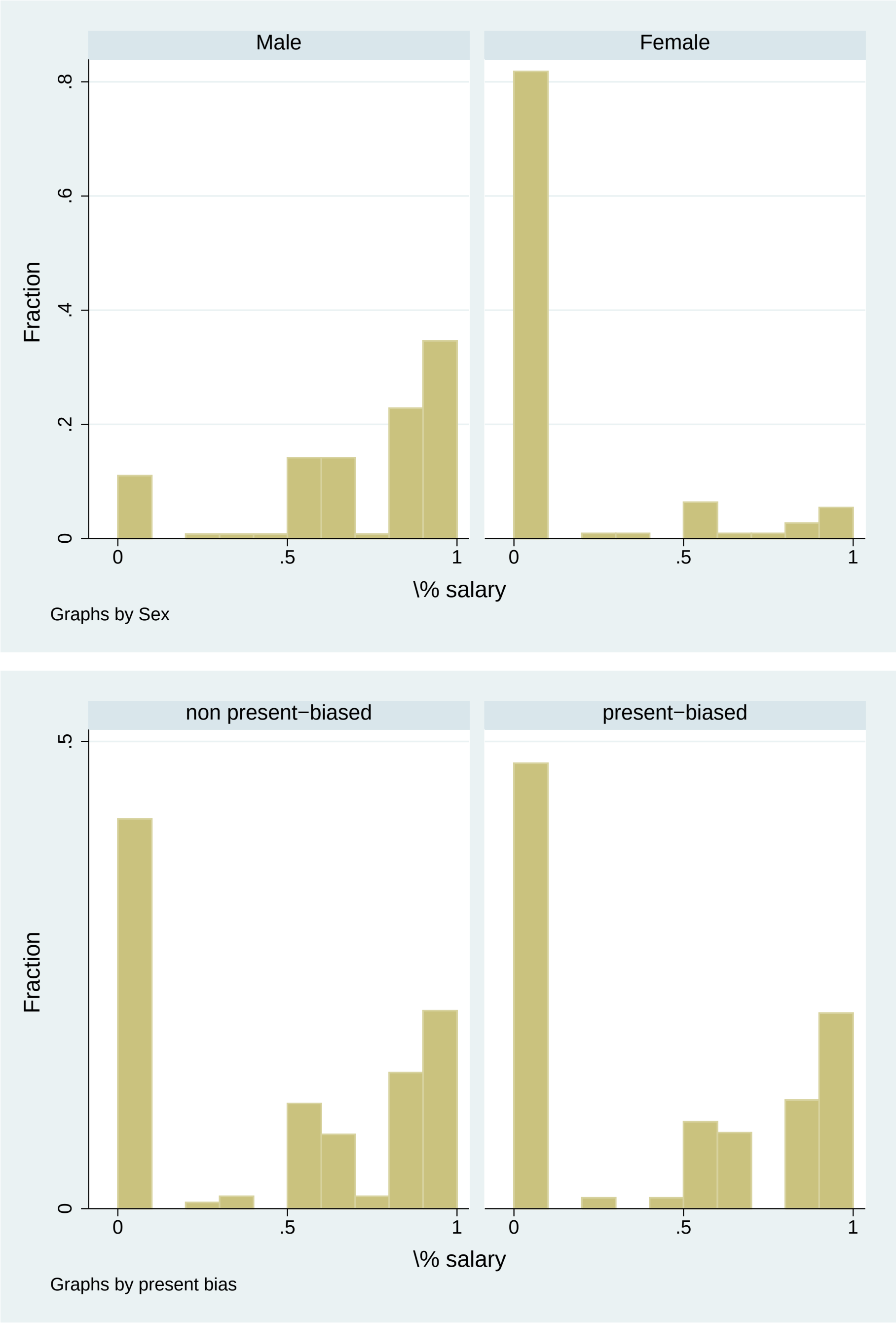

The share of earnings turned over to spouses is depicted for husbands and wives, and for non present-biased individuals and present-biased individuals.
