# Supplementary material for: Does marriage work as a savings commitment device? Experimental evidence from Vietnam
Source: PLoS One. 2019 Jun 19;14(6):e0217646. doi: 10.1371/journal.pone.0217646 (PMC6583950; doi:10.1371/journal.pone.0217646)
Supplement: S2 Fig — Estimated Kernel density function of the monthly allowance and monthly hidden disposal money are depicted. (PDF) [file pone.0217646.s002.pdf]

Supporting Information

S2 Fig. Kernel density of the monthly allowance and monthly disposal money without spouse's agreement

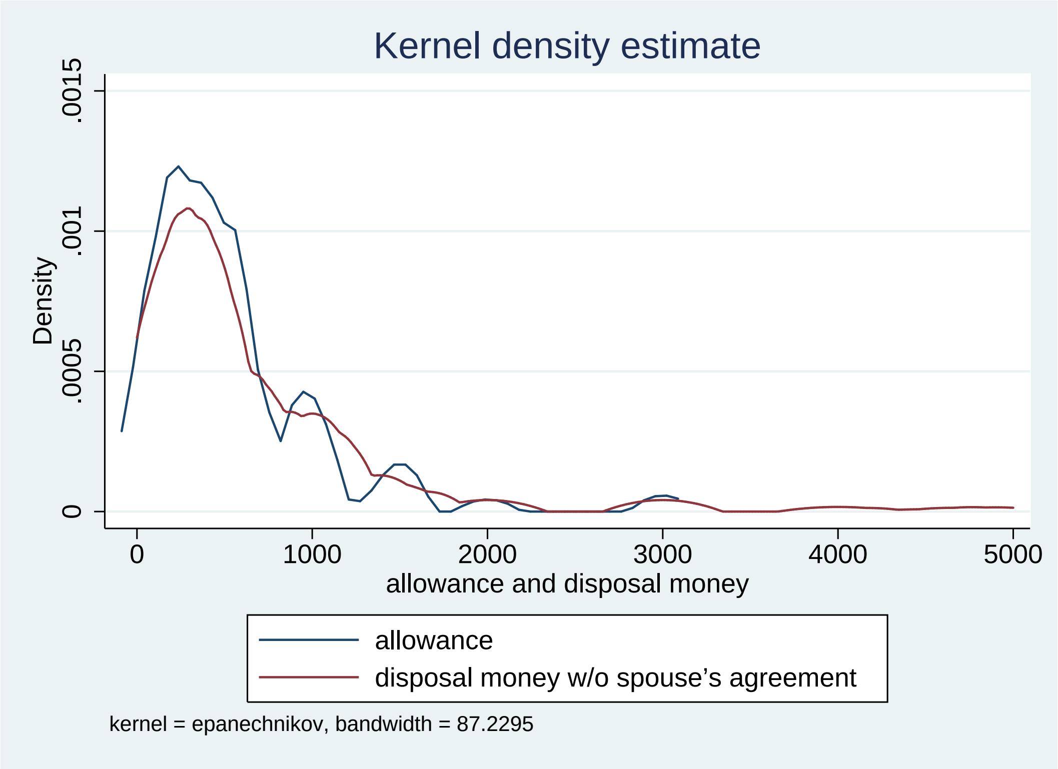

Estimated Kernel density function of the monthly allowance and monthly hidden disposal money are depicted, with using the Epanechnikov kernel and the optimal bandwidth.
