# Supplementary material for: Does marriage work as a savings commitment device? Experimental evidence from Vietnam
Source: PLoS One. 2019 Jun 19;14(6):e0217646. doi: 10.1371/journal.pone.0217646 (PMC6583950; doi:10.1371/journal.pone.0217646)
Supplement: S1 Table — This table reports the estimated average partial effects of the Tobit model. The control variables include a female dummy (if applicable) and standardized differences in income, assets, age, education, arithmetic score, and financial literacy. (PDF) [file pone.0217646.s003.pdf]

Supporting Information

S1 Table. The share of earnings turned over to spouses: Including interaction terms of PB and bargaining power

	(1) All	(2) Husb	(3) wife	(4) All	(5) Husb	(6) wife	(7) All	(8) Husb	(9) wife
PB & sp NPB	-0.327**	-0.255	-0.543	-0.345**	-0.272*	-0.548	-0.350**	-0.325*	-0.521
	(0.161)	(0.163)	(0.609)	(0.170)	(0.159)	(0.614)	(0.167)	(0.184)	(0.657)
NPB & sp PB	0.372**	0.246	1.589**	0.360**	0.210	1.297**	0.337**	0.108	1.470*
	(0.158)	(0.157)	(0.646)	(0.165)	(0.159)	(0.622)	(0.162)	(0.163)	(0.751)
PB & sp NPB× dif_asset	0.260**	0.228**	0.333						
	(0.111)	(0.091)	(0.594)						
NPB & sp PB× dif_asset	-0.050	-0.087	0.640						
	(0.120)	(0.144)	(0.452)						
PB & sp NPB× dif_edu				0.041	0.131	-0.959			
				(0.162)	(0.132)	(0.817)			
NPB & sp PB× dif_edu				0.006	-0.006	0.099			
				(0.163)	(0.110)	(0.526)			
PB & sp NPB× dif_age							0.087	0.099	0.151
							(0.153)	(0.161)	(0.529)
NPB & sp PB× dif_age							0.131	0.217*	0.202
							(0.141)	(0.117)	(0.741)
Observations	237	127	110	237	127	110	237	127	110

The estimated average partial effects of the Tobit model are reported. The control variables include a female dummy (if applicable) and standardized differences in income, assets, age, education, arithmetic score, and financial literacy. Standard errors clustered by couple are in parentheses. Asterisks indicate statistical significance: * $p < .10$, ** $p < .05$, *** $p < .01$.
