# Supplementary material for: Does marriage work as a savings commitment device? Experimental evidence from Vietnam
Source: PLoS One. 2019 Jun 19;14(6):e0217646. doi: 10.1371/journal.pone.0217646 (PMC6583950; doi:10.1371/journal.pone.0217646)
Supplement: S2 Table — This table reports the estimated coefficients of the linear probability model. The control variables include a female dummy (if applicable) and standardized differences in income, assets, age, education, arithmetic score, and financial literacy. (PDF) [file pone.0217646.s004.pdf]

S2 Table. Subjects who are financial managers in their households: Linear probability model

	(1) All	(2) All	(3) Husband	(4) Wife	(5) All	(6) Husband	(7) Wife
Present-biased (PB)	0.090	0.035	0.226**	-0.016	0.211**	0.433*	0.068
	(0.056)	(0.070)	(0.110)	(0.072)	(0.093)	(0.224)	(0.068)
Spouse is PB	-0.031	-0.021	-0.051	-0.019	-0.033	-0.044	-0.025
	(0.067)	(0.068)	(0.105)	(0.086)	(0.067)	(0.106)	(0.086)
PB & Joint decision non-PB		0.084					
		(0.080)					
Sophisticated PB					-0.141	-0.229	-0.099
					(0.094)	(0.225)	(0.074)
Observations	268	268	134	134	268	134	134

	(1) All	(2) Husband	(3) Wife	(4) All	(5) Husband	(6) Wife
PB & Spouse is not PB	0.083	0.253*	-0.042	0.296**	0.543**	0.072
	(0.065)	(0.139)	(0.089)	(0.130)	(0.263)	(0.094)
Not PB & Spouse is PB	-0.037	-0.029	-0.051	-0.033	-0.016	-0.052
	(0.070)	(0.116)	(0.103)	(0.070)	(0.116)	(0.104)
PB & Spouse is PB	0.063	0.160	-0.018	0.069	0.177	-0.017
	(0.071)	(0.159)	(0.115)	(0.070)	(0.160)	(0.115)
PB & Spouse is not PB				-0.236*	-0.316	-0.127
× sophisticated PB				(0.134)	(0.258)	(0.095)
Observations	268	134	134	268	134	134

This table reports the estimated coefficients of the linear probability model. We include the same control variables as in Table 4. Standard errors clustered by couple are in parentheses. Asterisks indicate statistical significance: * $p < .10$, ** $p < .05$, and *** $p < .01$.
