# Supplementary material for: Does marriage work as a savings commitment device? Experimental evidence from Vietnam
Source: PLoS One. 2019 Jun 19;14(6):e0217646. doi: 10.1371/journal.pone.0217646 (PMC6583950; doi:10.1371/journal.pone.0217646)
Supplement: S3 Table — This table reports the estimated coefficients of an ordinary least squares regression. The control variables include a female dummy (if applicable) and standardized differences in income, assets, age, education, arithmetic score, and financial literacy. (PDF) [file pone.0217646.s005.pdf]

Supporting Information

S3 Table. Allowance: Including the share of earnings turned over to the spouse

Monthly allowance:

	(1) All	(2) All	(3) husband	(4) wife	(5) husband	(6) wife
present bias(PB)	-189.690***	-58.481	-293.820**	-65.159	-249.648*	109.811
	(68.778)	(80.322)	(126.999)	(84.435)	(146.725)	(132.362)
spouse PB(sp PB)	-103.781	-130.177	48.531	-347.614**	40.530	-387.099**
	(84.084)	(85.521)	(89.043)	(159.258)	(93.096)	(159.442)
PB but joint NPB		-210.840**			-80.426	-267.877*
		(91.200)			(138.701)	(141.696)
% salary	-110.938	-113.221	-233.450	22.238	-244.092	59.339
	(104.245)	(105.196)	(160.208)	(121.325)	(164.059)	(126.278)
Observations	237	237	127	110	127	110

Hidden disposal money:

	(1) All	(2) All	(3) husband	(4) wife	(5) husband	(6) wife
present bias(PB)	169.315	57.163	66.129	246.585	90.863	-18.352
	(111.495)	(111.118)	(108.778)	(173.382)	(141.074)	(175.974)
spouse PB(sp PB)	-89.932	-67.370	19.539	-224.768	15.059	-164.981
	(123.149)	(116.155)	(79.952)	(303.377)	(81.327)	(283.432)
PB but joint NPB		180.218			-45.034	405.616
		(148.466)			(162.883)	(275.605)
% salary	-242.285**	-240.334**	-276.934	-228.089	-282.894	-284.266
	(102.828)	(106.172)	(183.240)	(160.607)	(194.423)	(177.943)
Observations	237	237	127	110	127	110

The estimated coefficients of an OLS regression are reported. The control variables include a female dummy (if applicable) and standardized differences in income, assets, age, education, arithmetic score, and financial literacy. Standard errors clustered by couple are in parentheses. $+$ and $*$ indicate $p < .10$ and $p < .05$, respectively.
