# Supplementary material for: Does marriage work as a savings commitment device? Experimental evidence from Vietnam
Source: PLoS One. 2019 Jun 19;14(6):e0217646. doi: 10.1371/journal.pone.0217646 (PMC6583950; doi:10.1371/journal.pone.0217646)
Supplement: S4 Table — This table reports the estimated coefficients of the linear probability model. The same control variables as those used in Table 3 are included. (PDF) [file pone.0217646.s006.pdf]

Supporting Information

S4 Table. Participation in ROSCAs: Linear probability model

	(1) All	(2) All	(3) All	(4) husband	(5) wife	(6) husband	(7) wife
Present-biased (PB)	0.023	-0.016		0.029	0.021		
	(0.049)	(0.065)		(0.089)	(0.072)		
Spouse is PB	0.037	0.045		0.034	0.082		
	(0.057)	(0.059)		(0.075)	(0.119)		
PB & Joint decision non-PB		0.061					
		(0.068)					
PB & Spouse is not PB			0.112*			0.129	0.102
			(0.064)			(0.102)	(0.082)
Not PB & Spouse is PB			0.126*			0.115	0.183
			(0.073)			(0.088)	(0.131)
PB & Spouse is PB			0.003			0.010	0.049
			(0.068)			(0.075)	(0.119)
Control	Yes	Yes	Yes	Yes	Yes	Yes	Yes
Observations	268	268	268	134	134	134	134

The table reports the estimated coefficients of the linear probability model. The same control variables as in Table 4 are included. Standard errors clustered by couple are in parentheses. Asterisks indicate statistical significance: * $p < .10$, ** $p < .05$, and *** $p < .01$.
