# Supplementary material for: Does marriage work as a savings commitment device? Experimental evidence from Vietnam
Source: PLoS One. 2019 Jun 19;14(6):e0217646. doi: 10.1371/journal.pone.0217646 (PMC6583950; doi:10.1371/journal.pone.0217646)
Supplement: S5 Table — This table reports estimated average partial effects of the probit model (savings) and an ordinary least squares (assets) regression. The same control variables as in Table 3 are included. The sample of husbands includes no observations in which both spouses are PB and the husband joined a ROSCA; therefore, these observations are dropped from the estimation, leaving us with 117 observations. (PDF) [file pone.0217646.s007.pdf]

Supporting Information

S5 Table. Savings and assets

	(1) Saving	(2) Saving	(3) Saving	(4) Asset	(5) Asset	(6) Asset
present bias(PB)	0.027	0.109		-64.286	79.583	
	(0.130)	(0.164)		(176.666)	(244.408)	
spouse PB(sp PB)	-0.008	-0.023		171.617	145.377	
	(0.121)	(0.121)		(124.571)	(126.628)	
PB but joint NPB		-0.142			-248.169	
		(0.161)			(254.017)	
PB & sp NPB			0.009			-68.852
			(0.149)			(206.142)
NPB & sp PB			-0.023			167.933
			(0.133)			(128.664)
PB & sp PB			0.029			109.807
			(0.180)			(240.533)
Control	Yes	Yes	Yes	Yes	Yes	Yes
Observations	134	134	134	134	134	134

The estimated average partial effects of the probit model (savings) and the ordinary least squares (assets) are reported. The same control variables as in Table **??** are included. For husbands sample, there are no observations in which both spouses are PB and who joined ROSCAs; therefore, these observations are dropped from the estimation, leaving us with 117 observations. Standard errors clustered by couple are in parentheses. Asterisks indicate statistical significance: * $p < .10$, ** $p < .05$, *** $p < .01$.
