# Supplementary material for: Does marriage work as a savings commitment device? Experimental evidence from Vietnam
Source: PLoS One. 2019 Jun 19;14(6):e0217646. doi: 10.1371/journal.pone.0217646 (PMC6583950; doi:10.1371/journal.pone.0217646)
Supplement: S6 Table — This table reports the result of the robustness check in which the subjects who never shifted or the subjects who joined ROSCAs were dropped from the estimation. (PDF) [file pone.0217646.s008.pdf]

Supporting Information

S6 Table. Robustness

Drop observations who never shift

	(1) salary	(2) salary:husb	(3) salary:wife	(4) keepcash	(5) keepcash
PB & sp NPB	-0.374**	-0.218	-0.585	0.030	0.328**
	(0.175)	(0.176)	(0.657)	(0.071)	(0.163)
NPB & sp PB	0.422*	0.316	1.528	-0.124	-0.109
	(0.239)	(0.203)	(0.963)	(0.079)	(0.077)
PB & sp PB	0.229	0.198	0.984	0.055	0.067
	(0.216)	(0.198)	(0.792)	(0.079)	(0.077)
PB & sp NPB× soph PB					-0.322*
					(0.167)
Observations	150	76	74	169	169

	(1) allowance	(2) allowance	(3) hidden money	(4) hidden money	(5) rosca	(6) rosca:wife
present bias(PB)	-161.395***	-73.160	227.249**	57.232		
	(60.675)	(86.718)	(98.439)	(99.650)		
spouse PB(sp PB)	29.243	0.978	-111.284	-56.822		
	(74.125)	(77.492)	(141.803)	(130.148)		
PB but joint NPB		-135.854		261.770**		
		(86.997)		(118.852)		
PB & sp NPB					0.110*	0.040
					(0.058)	(0.072)
NPB & sp PB					0.165**	0.170
					(0.072)	(0.109)
PB & sp PB					0.003	0.032
					(0.093)	(0.123)
Observations	169	169	169	169	169	88

Drop observations who join ROSCAs

	(1) salary	(2) salary:husb	(3) salary:wife	(4) keepcash	(5) keepcash
PB & sp NPB	-0.239	-0.171	-0.239	0.061	0.410**
	(0.150)	(0.156)	(0.554)	(0.069)	(0.180)
NPB & sp PB	0.343**	0.173	1.667***	-0.007	0.007
	(0.168)	(0.165)	(0.642)	(0.070)	(0.068)
PB & sp PB	0.233	0.117	1.401*	0.092	0.106
	(0.189)	(0.180)	(0.715)	(0.071)	(0.069)
PB & sp NPB× soph PB					-0.381**
					(0.184)
Observations	212	117	95	240	240
